# In this issue

**Published:** 2023-03

**The expected economic burden on the healthcare system because of quarantining patients with monkeypox virus**

Alshahrani determines the expected economic cost for healthcare systems due to quarantine in the case of the monkeypox virus. A systematic literature review of studies on similar virus outbreaks was performed. The findings affirm that quarantine effectively mitigates the spread of a virus outbreak, but it has high direct and indirect costs that can only be justifiable for a dangerous virus with high mortality. The monkeypox virus presents moderate risk, unlike high-risk diseases for which quarantine is mandatory. The study recommends the introduction of mass vaccination programs and public awareness and sensitization forums to inform the population about the best behavioral practices to curb the spread of monkeypox virus.

***see page 231***

