# Supplementary material for: In this issue
Source: Saudi Med J. 2023 Mar;44(3):229. (PMC10043899)
Supplement: Related texts

# Abstract: Original Articles

**Figure:** The results of receiver operating characteristic curve analysis for C-reactive protein, D-dimer, ferritin, and neutrophil/lymphocyte ratio.

**COVID-19 infection during pregnancy. *A retrospective study in a city in the Southeastern region of Turkey***

Akgül et al evaluate maternal and fetal clinical outcomes in SARS-CoV-2 infected pregnant women during the whole period of the pandemic in a single province in the southeast of Turkey. They concluded that in the 3rd trimester of pregnancy, obesity and hypothyroidism were found to be risk factors for severe-critical cases of COVID-19 infection, and the clinical course was more severe with a higher rate of mortality in the recent period of the pandemic.

***see page 268***

**Self-care practices among heart failure patients. *A cross-sectional study in Saudi population***

Aljohani describes self-care practices among Saudi heart failure (HF) patients and identify sociodemographic characteristics contributing to self-care practices. He concluded that Self-care practices in this study showed higher scores than those reported in international studies. Further studies are warranted to explore everyday self-care needs and challenges among HF patients.

***see page 277***

**Patellar height changes after treatment of tibia plateau fractures. A radiological analysis**

**Figure:** Sample measurements of patellar height index values are seen on the X-ray of the patient who underwent lateral plate due to tibial plateau fracture.

Doğan et al determine the impact of tibial plateau fractures on patellar height and the factors affecting this impact in in a retrospective prognostic study. They concluded that Long-term functions of tibial plateau fractures should be evaluated not only with a painless range of motion but also with patellar height. It should be noted that the Luo classification, which evaluates the plateau 3-dimensionally, may be associated with changes in postoperative patellar height values

***see page 306***
